# Antimicrobial potential and rhodamine B dye degradation using graphitic carbon nitride and polyvinylpyrrolidone doped bismuth tungstate supported with in silico molecular docking studies

**DOI:** 10.1038/s41598-023-44799-9

**Published:** 2023-10-19

**Authors:** Muhammad Hasnain Ashfaq, Muhammad Imran, Ali Haider, Anum Shahzadi, Muhammad Mustajab, Anwar Ul-Hamid, Walid Nabgan, Francisco Medina, Muhammad Ikram

**Affiliations:** 1 Department of Chemistry, Government College University, Sahiwal Road, Sahiwal, Faisalabad, 57000 Punjab Pakistan; 2 Department of Clinical Sciences, Faculty of Veterinary and Animal Sciences, Muhammad Nawaz Shareef, University of Agriculture, Multan, 66000 Punjab Pakistan; 3 Department of Pharmacy, COMSATS University Islamabad, Lahore Campus, Lahore, 54000 Pakistan; 4 Solar Cell Applications Research Lab, Department of Physics, Government College University Lahore, Lahore, 54000 Punjab Pakistan; 5 Center for Engineering Research, Research Institute, King Fahd University of Petroleum and Minerals, 31261 Dhahran, Saudi Arabia; 6 Departament d’Enginyeria Química, Universitat Rovira I Virgili, Av Països Catalans 26, 43007 Tarragona, Spain

**Keywords:** Analytical chemistry, Catalysis

## Abstract

The environmental-friendly hydrothermal method has been carried out to synthesize Bi_2_WO_6_ and g-C_3_N_4_/PVP doped Bi_2_WO_6_ nanorods (NRs) by incorporating different concentrations of graphitic carbon nitride (g-C_3_N_4_) as well as a specified quantity of polyvinylpyrrolidone (PVP). Bi_2_WO_6_ doped with g-C_3_N_4_ provides structural and chemical stability, reduces charge carriers, degrades dyes, and, owing to lower bandgap energy, is effective for antibacterial, catalytic activity, and molecular docking analysis. The purpose of this research is the treatment of polluted water and to investigate the bactericidal behavior of a ternary system. The catalytic degradation was performed to remove the harmful rhodamine B (RhB) dye using NaBH_4_ in conjunction with prepared NRs. The specimen compound demonstrated antibacterial activity against Escherichia coli (E. coli) at both high and low concentrations*.* Higher doped specimens of g-C_3_N_4_/PVP-doped Bi_2_WO_6_ exhibited a significant improvement in efficient bactericidal potential against *E. coli* (4.55 mm inhibition zone). In silico experiments were carried out on enoyl-[acylcarrier-protein] reductase (FabI) and β-lactamase enzyme for *E. coli* to assess the potential of Bi_2_WO_6_, PVP doped Bi_2_WO_6_, and g-C_3_N_4_/PVP-doped Bi_2_WO_6_ NRs as their inhibitors and to justify their possible mechanism of action.

## Introduction

Water is a naturally occurring and vital source for the survival of living organisms on the earth and the natural food chain^1^. Population growth limits the access of many people to freshwater^2^. Water bodies are being polluted by municipal sewage water, agricultural run-off, poor sanitation, animal waste, hospital septic system run-off, and many harmful dyes and hazardous chemicals released by industries including paper, rubber, textile, pharmaceutical, cosmetics, leather, etc. into the water reservoirs^3^. Organic dyes are challenging to break down under natural circumstances owing to their stable and complex chemical compositions designed to withstand degradation by light, chemical, biological and other factors. Rhodamine B (RhB) is a significant amino xanthene dye extensively used in the textile industry, biological and fluorescent stain industries, colored glass sector, and the pyrotechnics industry^4^. The International Agency for Research on Cancer categorized the RhB as a Group 3 carcinogenic compound^5^. RhB causes teratogenicity, carcinogenicity, and mutagenicity in human and animal cells and is nevertheless recognized to be toxic^6^. Furthermore, bacteriological species *Escherichia coli (E. coli)*, *Methicillin-resistant Staphylococcus aureus* (MRSA), *Bacillus subtilis*, *Staphylococcus aureus (S. aureus)*, and *Pseudomonas* are present in sewage, animal waste, and septic wastewater leading to many diseases like typhoid fever, cholera, diarrhea, and bacillary dysentery. According to an estimate by the Infectious Diseases Society of America (IDSA), 0.13 billion children under five die due to diarrhea, primarily spread by polluted water containing *E. coli*^7^. These extremely hazardous contaminants cause serious health problems to marine life and human cancer, allergies, skin irascibilities, liver diseases, eye irritability, respiratory tract irritation, skin irritation, and cornea in rabbits^8^. In this regard, a variety of techniques have been deployed to purify industrially contaminated water, including membrane filtration^9^, catalytic degradation and photocatalytic^10,11^ electrolysis^12^, solvent extraction^13^ and adsorption^14^. Nevertheless, commonly used approaches still have a lot of drawbacks, such as high levels of energy consumption, low levels of efficiency in terms of degradation, secondary pollution due to inadequate removal, and dye transfer^15^. The photocatalytic and catalytic activity of nanomaterials (NMs) were used as water purification techniques offering ecological compatibility, inexpensiveness, and energy efficiency. Catalytic activity (CA) is applied to remove hazardous inorganic and organic pollutants from effluent water due to low-cost and energy efficiency compared to other techniques^16^.

NMs are characterized by high levels of reactivity, catalytic activity, and adsorption, all of which can be attributed to their small size and large surface area^17^. Many scientists are currently focusing their efforts on the development of photoactive materials such as BiVO_4_^18^, ZrP_2_O_7_^19^, and Bi_2_WO_6_^20,21^. Among these, bismuth-based materials have been recognized as significant materials and sustained worthy attention for wastewater purification because of the smaller band gap. Bi_2_WO_6_ has a large surface area and bulk energy gap that are effective tool for rapidly reducing pollutants. Because of high photocatalytic activity and ability to split water when exposed to visible light, Bi_2_WO_6_ has garnered a lot of attention recently due to ionic conductivity and catalytic activity^20,22^.

Amorphous, non-toxic, highly soluble, and non-ionic characteristics of a synthetic polymer such as polyvinylpyrrolidone (PVP) have received a lot of attention in wastewater treatment^23^. PVP is hydrophilic, and can influence the morphological characteristics and development of NMs by ensuring their solubility in a variety of solvents, exceptional stability to molecules, governing the development of crystal structure, functioning as a shape-regulator, and serving as a capping agent to control the size of Bi_2_WO_6_. PVP molecules combined with functional groups C=O, C–N, and CH_2_ are the most common molecules used to remove organic contaminants from wastewater^24,25^. The addition of PVP enhances the stability of the particle through the enhancement of steric stabilization and the inhibition of particle growth. The fixed PVP concentration has the capability of regulating particle size to achieve optimal average sizes. As the concentration of PVP increased, a stable zone was observed wherein particle size exhibited no significant variations. The manipulation of PVP concentration enables the regulation and enhancement of various characteristics of nanoparticles, including their structure, morphology, size, composition, and optical properties^26^. Researchers have been interested in 2D materials graphene oxide (GO), molybdenum disulfide (MoS_2_), and graphitic carbon nitride (g-C_3_N_4_). Among these materials, g-C_3_N_4_ has garnered the most attention. This is due to its large specific surface area, good electron conductivity, high adsorption capacity, and promising band gap (2.7 eV). g-C_3_N_4_ is nontoxic, biocompatible, and non-metallic compound^27^.

The previous study examined the catalytic decolorization of a mixture solution of RhB using the synthesized material under experimental conditions, in the absence of light. The catalytic characteristics of g-C_3_N_4_/ZnO resulted in exceptional dye degradation. The catalytic properties of the g-C_3_N_4_/ZnO composite exhibited significant enhancement compared to pure g-C_3_N_4_. Notably, the catalytic activity improved progressively with increasing concentration of g-C_3_N_4_. However, at higher levels of g-C_3_N_4,_ a slight decline in catalytic properties was observed. This indicates that the optimal concentration of g-C_3_N_4_ in the g-C_3_N_4_/ZnO composites was determined to be 5.0 wt%. In 100 min, the (5.0 wt%) g-C_3_N_4_/ZnO catalyst exhibited a 97.4% oxidation efficiency towards RhB, while demonstrating a 75.5% reduction efficiency. The proposal suggests that the number of trapping sites for carriers is enhanced when the quantity of g-C_3_N_4_ increases up to 5.0 wt%. This increase in trapping sites leads to a longer lifetime for carriers, ultimately resulting in improved catalytic activity. However, when the concentration of g-C_3_N_4_ exceeds 5.0 wt%, the presence of a high-concentration dopant can function as a recombination center for both electrons and holes, leading to a reduction in catalytic activity so that maximum catalytic degradation has been achieved in optimum amount of doped sample that is less than 5.0 wt%^28^.

This research aimed to investigate the influence of fixed concentration of PVP concentration and varying concentrations (2, 4 wt%) of g-C_3_N_4_ have an impact on the Bi_2_WO_6_ NRs that are produced using the hydrothermal method. The effect of g-C_3_N_4_/PVP-doped Bi_2_WO_6_ was investigated by studying the catalytic degradation behavior, antimicrobial activity, molecular docking analysis, and various characterization techniques to investigate the morphological, optical, and structural properties.

## Experimental section

### Materials

Bismuth nitrate pentahydrate (Bi_2_(NO_3_)_3_.5H_2_O) was purchased from BDH Laboratory Supplies, sodium tungstate dihydrate (Na_2_WO_4_. 2H_2_O), polyvinylpyrrolidone (PVP), acetic acid (CH_3_COOH), urea (CH_4_N_2_O) were purchased from Sigma-Aldrich. There was no need for additional purification because all the chemicals were of an analytical grade.

### Synthesis of graphitic carbon nitride

g-C_3_N_4_ has been prepared in the laboratory by pyrolysis of urea at 550 °C for 2 h. A dirty yellow-colored product was obtained that confirmed the synthesis of g-C_3_N_4_.

### Synthesis of bismuth tungstate and g-C_3_N_4_/PVP-doped Bi_2_WO_6_

Bi_2_WO_6_ and g-C_3_N_4_/PVP-doped Bi_2_WO_6_ nanorods (NRs) were prepared using a simple and environmentally friendly hydrothermal method. Initially, 0.5 M Bi_2_(NO_3_)_3_.5H_2_O was dissolved in 40 mL deionized water (DIW) and 16 mL of CH_3_COOH, marked as solution A. Furthermore, 0.25 M of Na_2_WO_4_.2H_2_O was dissolved in 24 mL DIW, marked as solution B. Solution B was added to solution A dropwise and placed under constant stirring for 30 min to acquire Bi_2_WO_6_. Afterward, (3%) PVP and g-C_3_N_4_ with particular concentrations (2 and 4 wt%) were incorporated into the pristine solution. Consequently, the solutions were added to a 100 mL Teflon-lined hydrothermal reactor (autoclave) in an oven at 180 °C for 24 h. The resulting products were centrifuged at 7000 rpm for 7 min with DIW and absolute ethanol to make the product free from impurities. The subsequent product was placed on a hot plate under constant stirring at 100 °C until the water evaporated thoroughly. The dried products were ground to sustain the fine powder for further characterization.

### Catalytic activity

To determine how much RhB dye is degraded, the investigators examined the catalytic activity (CA) of pristine and g-C_3_N_4_/PVP-doped Bi_2_WO_6_ NRs. The dye is broken down through a series of oxidation and reduction processes during the redox reaction that is part of the catalytic degradation process. Catalysis activity was used to determine the efficacy of synthetic dyes for degradation in the presence of sodium borohydride (NaBH_4_) and the synthesized nanocatalyst. RhB and NaBH_4_ were both freshly prepared before being used in the catalytic reactions to ensure that the data from the experiments were accurate. 3 mL of RhB dye was added to a mixture containing 400 µL of NaBH_4_ and 400 µL of synthesized NRs as a catalyst. The primary function of a catalyst has been used to reduce the activation energy (Ea) of a reaction, which ultimately increases both the reaction’s rate and its degree of stability. The surface activation energy of the reaction has been reduced by using a catalyst, which has increased both the reaction’s stability and its effectiveness. A UV–Vis spectrophotometer was used to perform spectrophotometric measurements at regular intervals to obtain absorption variation spectra. A reduction of RhB took place, bringing about a color transformation from pink to colorless. % Degradation was calculated using the formula, % degradation = (C_o_ − C_t_)/C_o_ × 100, where C_o_ is the initial and C_t_ is the final absorbance at a specific time after the incorporation of materials.

### Isolation and identification of MDR *E. coli*

#### Sample collection and isolation

Direct milking into sterile glassware collected raw milk samples from lactating cows marketed at different markets, including veterinary hospitals and farms in Punjab, Pakistan. The raw milk samples were promptly transported to the laboratory at 4 °C temperature. MacConkey agar was utilized to count the number of coliform bacteria that were present in the raw milk. To isolate the bacteria, all plates were incubated at 37 °C for 48 h.

#### Identification and characterization of bacterial isolates

The first identification of *E. coli* was made by Bergey’s Manual of Determinative Bacteriology using colonial morphology, Gram stain, and many different biochemical tests^29^.

##### Antibiotic susceptibility

The disk diffusion method was utilized to perform the antibiotic susceptibility test, and Mueller Hinton agar (MHA) was the medium preferred for the microbial test^30^. The experiment was carried out to determine whether or not *E. coli* was resistant to the following antibiotics (classes): Gentamicin (CN) 10 µg (Aminoglycosides), Ceftriaxone (CRO) 30 µg (Cephalosporins), Amoxycillin (AX) 25 µg (Penicillin), Imipenem (IPM) 10 µg Carbapenem), Tetracycline (TE) 30 µg (Tetracyclines), Azithromycin (AZM) 15 µg (Macrolides), Ciprofloxacin (CIP) 5 µg (Quinolones), Septran (SXT) 25 µg and Augmentin (AMC) 30 µg^31^. The purified Cultures of *E. coli* had been established and brought to a turbidity of 0.5 MacFarland. They were spread-plated on MHA (Oxoid Limited, Basingstoke, UK), and the antibiotic disks were positioned sufficiently separated from one another on the surface of the inoculated plate to avoid overlying the zones of inhibition. The Clinical and Laboratory Standard Institute^32^ evaluated the results after 24 h of incubation on plates at 37 °C. MDR was a designation given to bacteria that tested positive for resistance to at least three drugs^33^.

##### Antimicrobial activity

The in vitro antibacterial activity of pristine and (2, 4%) g-C_3_N_4_/PVP-doped Bi_2_WO_6_ has been assessed via the agar well diffusion method on 10 characteristic isolates of MDR *E. coli* collected from mastitis milk. These isolates were tested on 10 different mastitis milk samples. Petri dishes have been inoculated with MDR *E. coli* at a concentration of 1.5 × 10^8^ CFU/mL (0.5 McFarland standard) using MacConkey agar. The 6 mm wells have been drilled using a sterile cork borer. The low and high concentrations of synthetically produced pristine and (2, 4%) g-C_3_N_4_/PVP-doped Bi_2_WO_6_ were utilized as (0.5 mg/50 µL) and (1.0 mg/50 µL) respectively. Ciprofloxacin at a concentration of (0.005 mg/50 µL) was used as the positive control^34^, while DIW (50 µL) was used as the negative control^35^.

##### Statistical analysis

One-way analysis of variance (ANOVA) was performed in SPSS 20^36^ using the inhibition zone as the unit of measurement for antimicrobial efficacy. Inhibition zone diameters were analyzed, and the results were declared statistically significant at *p* < 0.05.

### Molecular docking analysis

Molecular docking simulations were performed on enoyl- [acylcarrier-protein] reductase (FabI) and β-lactamase enzyme since both are necessary for bacterial survival^37^. The 3D structural dimensions of chosen enzyme targets were obtained from the protein data source (https://www.rcsb.org/), with the PDB Codes 4D46 for FabI_*E.coli*_^38^ and 4KZ6 for β-lactamase_*E. coli*_^39^. SYBYL-X 2.0 software was used for the docking analysis as reported in our previous studies^40,41^. The Sybyl-X2.0/SKETCH module was used to generate three-dimensional structures of specific compounds. Subsequently, energy reduction was performed using the Tripos force fieldb by employing Gasteiger Hückel atomic charges. The Surflex-Dock module, a component of the SYBYL-X 2.0 molecular modelling software package, was used to conduct flexible molecular docking simulations. These simulations aimed to investigate the binding interactions between nanoparticles and the active site residues of certain proteins. Hydrogens that were absent were incorporated. In accordance with the AMBER 7 FF99 force field, the allocation of atomic types and the application of atomic charges were performed. Ultimately, the Powell technique was used, using a convergence gradient of 0.5 kcal/ (mol・A) over a span of 1000 cycles, in order to mitigate steric conflicts and effectively minimize the energy. In each ligand-receptor complex system, a minimum of 20 highly refined docked postures were definitively preserved. The Hammerhead scoring system was used to evaluate the optimal putative ligand poses. The Surflex dock module utilizes an empirically derived consensus scoring function called cScore to generate and rank potential poses of ligand fragments. This scoring function combines various empirical scoring components, including D-score (dock score), G-score (gold score), Chem-Score, potential mean force (PMF) score, and/or complete score. Additionally, the module incorporates a molecular similarity method known as morphological similarity.

## Results and discussion

**Figure 1:**
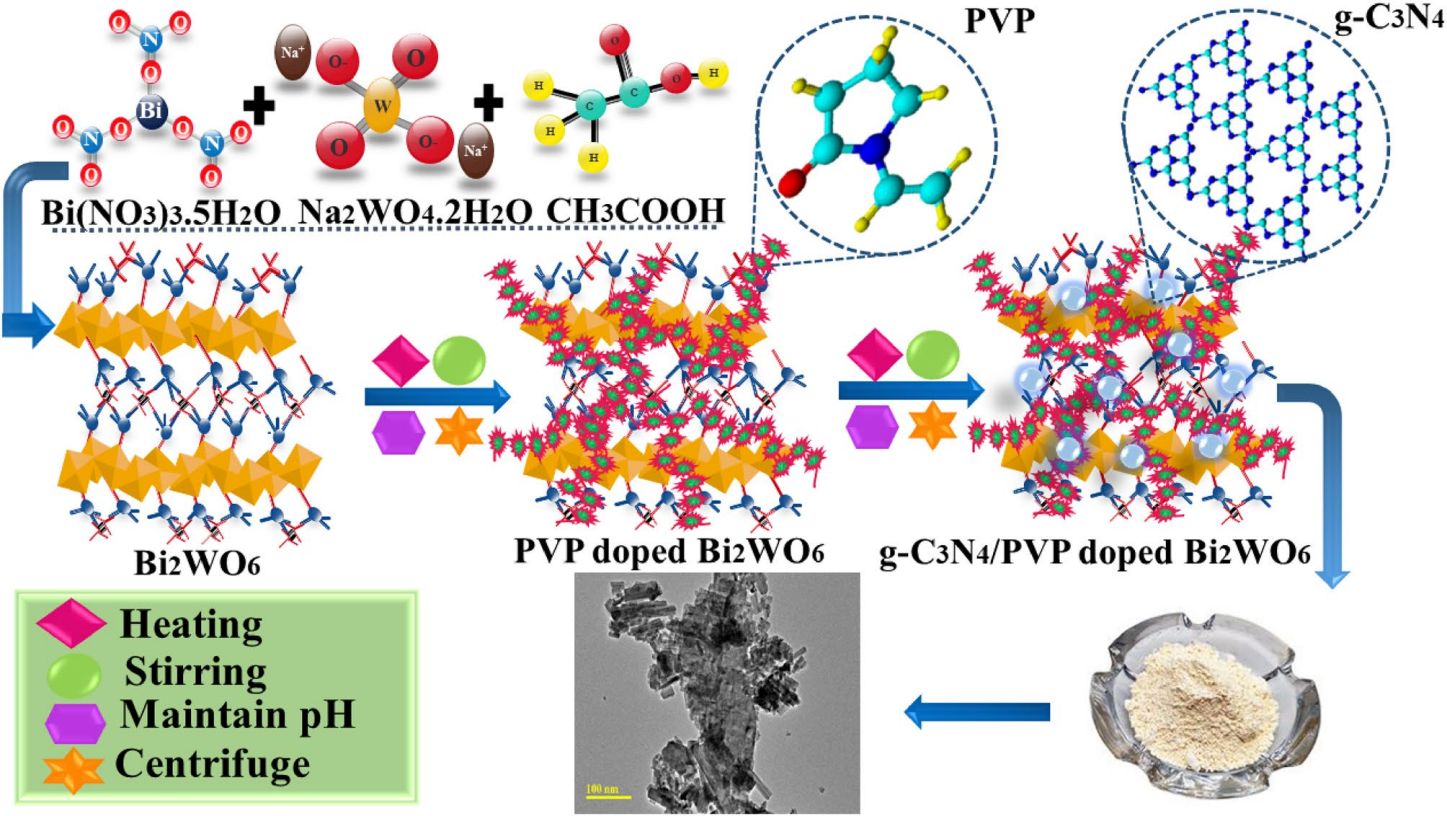
Schematic illustration of synthesized Bi_2_WO_6_ and g-C_3_N_4_/PVP-doped Bi_2_WO_6_.

The Bi_2_WO_6_ and g-C_3_N_4_/PVP-doped Bi_2_WO_6_ NRs have been synthesized using the hydrothermal method. g-C_3_N_4_ has been prepared in the laboratory by pyrolysis of urea (CH_4_N_2_O). Then various concentrations of g-C_3_N_4_ were incorporated in PVP-doped Bi_2_WO_6_ (Fig. 1).

**Figure 2:**
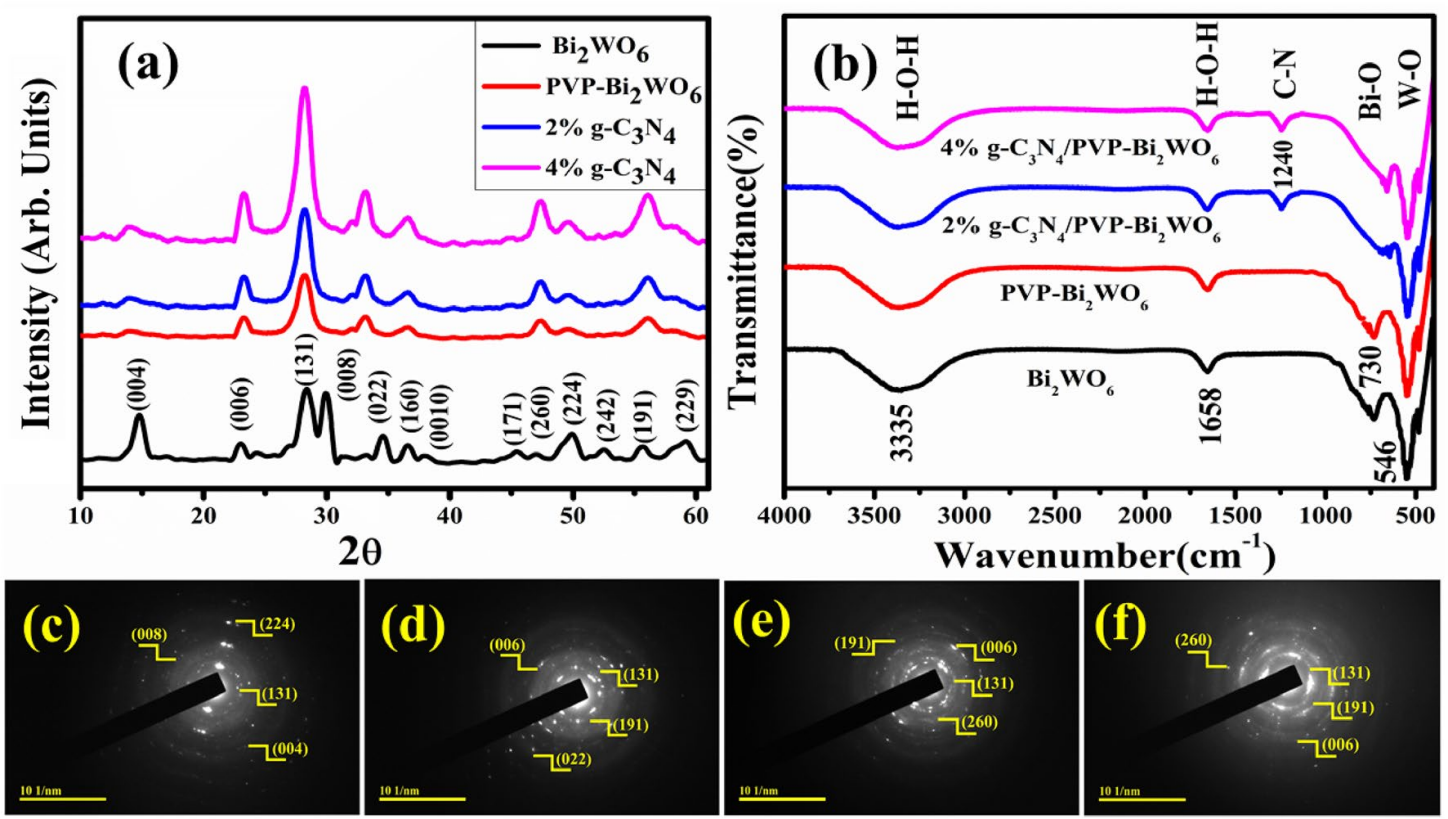
(**a**) X-ray diffraction spectra (**b**) FTIR spectra (**c**–**f**) SAED images of pristine and g-C_3_N_4_/PVP-doped Bi_2_WO_6_ NRs.

The constructive nature, crystal structures, phase purities, crystallinity, and crystal size of pristine and various (2, 4%) g-C_3_N_4_/PVP-doped Bi_2_WO_6_ NRs were examined through XRD analysis that has been conducted in the 2*θ* mode as represented in Fig. 2a. The results were analyzed with X-Pert High score software, which proclaimed that the unit cell of synthesized NRs has an orthorhombic structure with Pna21 and Pca21 and space group numbers 33 and 29. The two phases of bismuth tungstate oxide have been confirmed, which are Bi_2_WO_6_ and Bi_2_W_2_O_9_. The pristine sample (Bi_2_WO_6_) exhibits the diffraction peaks at 28.3°, 34.6°, 36.7°,45.2°, 47.0°, 52.4°, and 55.6° correspond to crystal planes (131), (022), (160), (171), (260), (242) and (191) respectively are well matched with the JCPDS card number 01-079-2381. Bi_2_W_2_O_9_ exhibits the diffraction peaks at 14.9°, 22.5°, 30.0°,37.9°, 49.9°, and 59.5° correspond to crystal planes (004), (006), (008), (0010), (224) and (229) respectively nicely coincide with JCPDS card number 01-089-8114. The crystalline size (D) of the synthesized NRs was estimated using the standard Debay-Scherrer relation D = 0.89λ/βcosθ Where K is a Scherrer’s Constant (0.94), λ is the wavelength (λ = 1.5418 Å) of X-ray radiation used and β is full width at half maximum (FWHM) of peaks at a different diffraction angle (2θ). For pristine and g-C_3_N_4_/PVP-doped Bi_2_WO_6_ NRs the crystallite size's measured value was 10.36, 10.34, 9.06, and 9.01 nm with corresponding (131) planes, respectively. Crystalline structures can be significantly altered in size by many factors, including the angle of diffraction, the effect of peak broadening, and other factors.

To understand the chemical structures and functional groups of synthesized Bi_2_WO_6_ and (2 and 4%) g-C_3_N_4_/PVP-doped Bi_2_WO_6_ NRs. The FT-IR spectroscopy revealed many bands that announced the presence of functional groups in the frequency range of 4000–400 cm^−1^, as represented in Fig. 2b. The bending vibrations of O–H are responsible for the stronger band observed, with the peak intensity at approximately 3335 cm^−1^. The presence of moisture in the atmosphere is responsible for the appearance of a weak band with a peak intensity of 1658 cm^−1^. These characteristic absorption peaks are assigned to the bending vibration of the H–O–H band^42^. The band appeared at 546 cm^−1^, associated with the stretching vibration of the W–O and the W–O–W^43^. The band found at 730 cm^−1^ belongs to the stretching vibration of the Bi-O^43^. The observed band confirms the presence of the synthesized material with the incorporation of g-C_3_N_4;_ a peak observed at 1240 cm^−1^ has been attributed to C-N, to confirm the presence of doped g-C_3_N_4_/PVP-doped Bi_2_WO_6_^44^.

The SAED patterns of both the pristine and the (2 and 4%) g-C_3_N_4_//PVP- Bi_2_WO_6_ NRs exhibit a distinguishable bright spot and a bright ring, as shown in Fig. 2c–f. The pristine Bi_2_WO_6_ points to the bright spots, laid out in a systematic pattern and taken up of individual crystals. Bright spot combinations generate bright rings of g-C_3_N_4_/PVP- Bi_2_WO_6_ NRs, which strongly indicates the polycrystalline nature of the synthesized materials. The rings offered indisputable evidence for pristine and doped NRs, have a highly crystalline composition, and the rings were aligned with the XRD observations.

**Figure 3:**
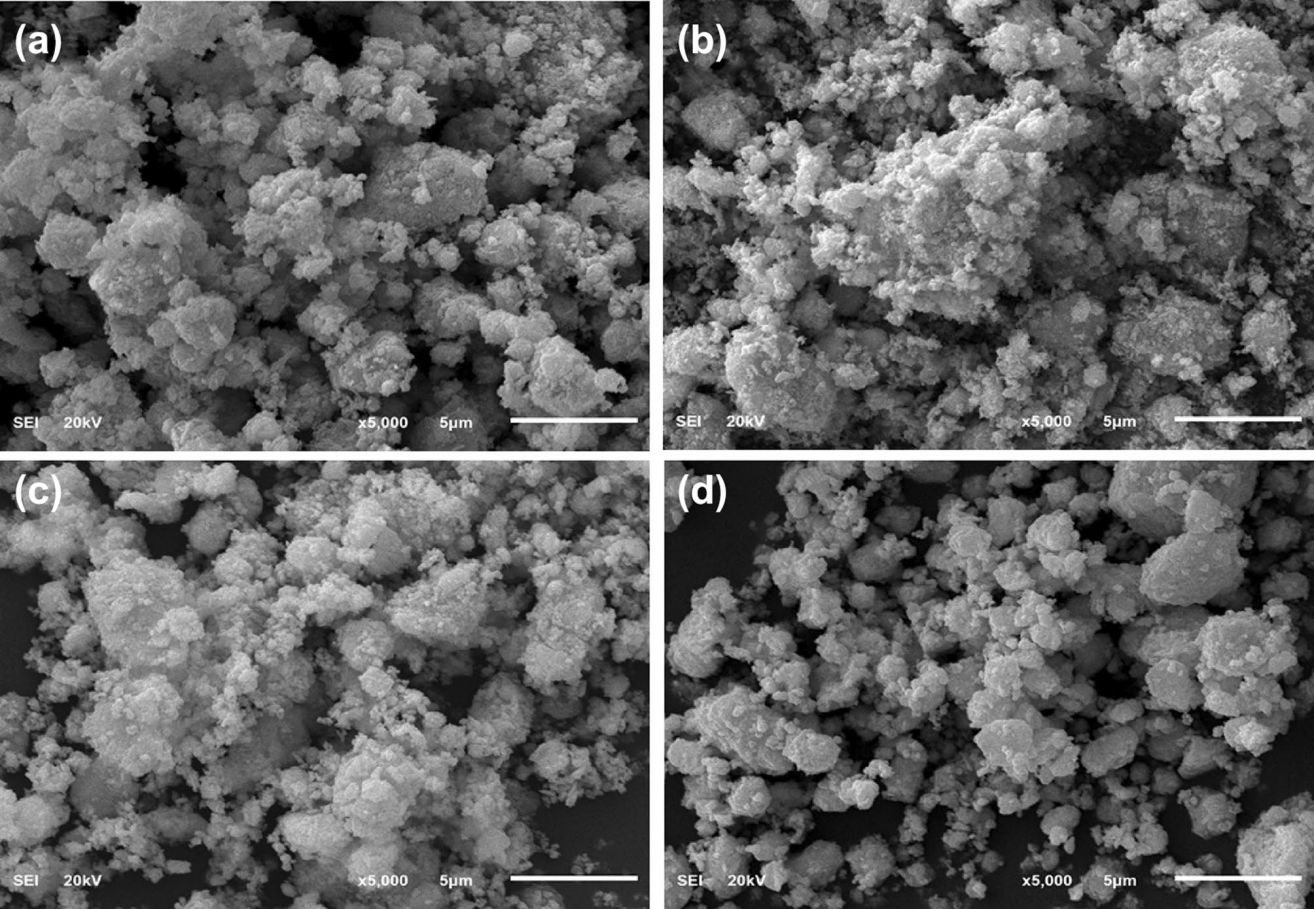
FESEM images of (**a**) Bi_2_WO_6_ (**b**) PVP-doped Bi_2_WO_6_ (**c**) 2% g-C_3_N_4_/PVP-doped Bi_2_WO_6_ NRs (**d**) 4% g-C_3_N_4_/PVP-doped Bi_2_WO_6_ NRs.

SEM micrographs have been used to identify the surface morphology of the prepared specimen indicated in Fig. 3a–d. Pristine samples indicate the agglomeration and aggregation of nanorods (NRs), and the NRs exhibit chunk-like morphology. Incorporating PVP in the control sample showed the overlapping of PVP around nanorods because PVP acts as a capping agent and also reduces the size and increases the surface area. The addition of various concentrations of g-C_3_N_4_ into the binary system PVP- Bi_2_WO_6_ represented the overlapping of 2D material (g-C_3_N_4_) to nanorods and nanoparticles, and accumulation increased with increasing amounts of g-C_3_N_4_.

**Figure 4:**
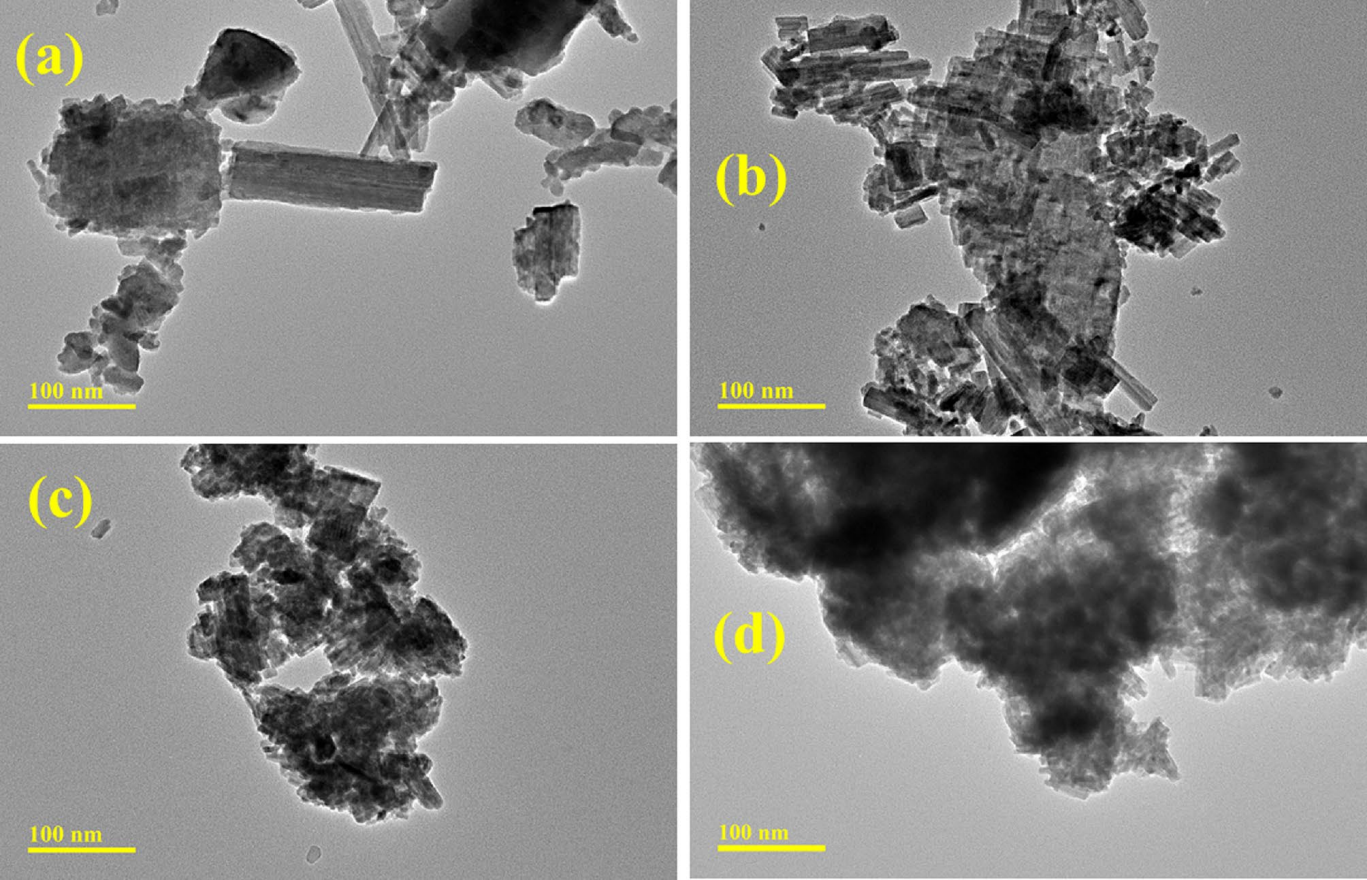
TEM images of (**a**) Bi_2_WO_6_ (**b**) PVP-doped Bi_2_WO_6_ (**c**) 2% g-C_3_N_4_/PVP-doped NRs (**d**) 4% g-C_3_N_4_/PVP-doped Bi_2_WO_6_ NRs.

The topography and morphology of the synthesized materials shown in Fig. 4a–d were determined through TEM analysis. The pristine sample reveals a rod-like morphology characterized by aggregation and agglomeration. Incorporating polymer (PVP), a function of the capping agent, demonstrates that the nanorods (NRs) have been capped. The incorporation of PVP indicates a decrease in agglomeration and an increase in aggregation due to the formation of covalent bonds or metallic bonds that are difficult to break. Incorporating g-C_3_N_4_/PVP increased the agglomeration of NRs, leading to an increase in surface area and a reduction in the sizes of the NRs. The surface became non-uniform as the doping increased gradually, and NRs started to appear.

**Figure 5:**
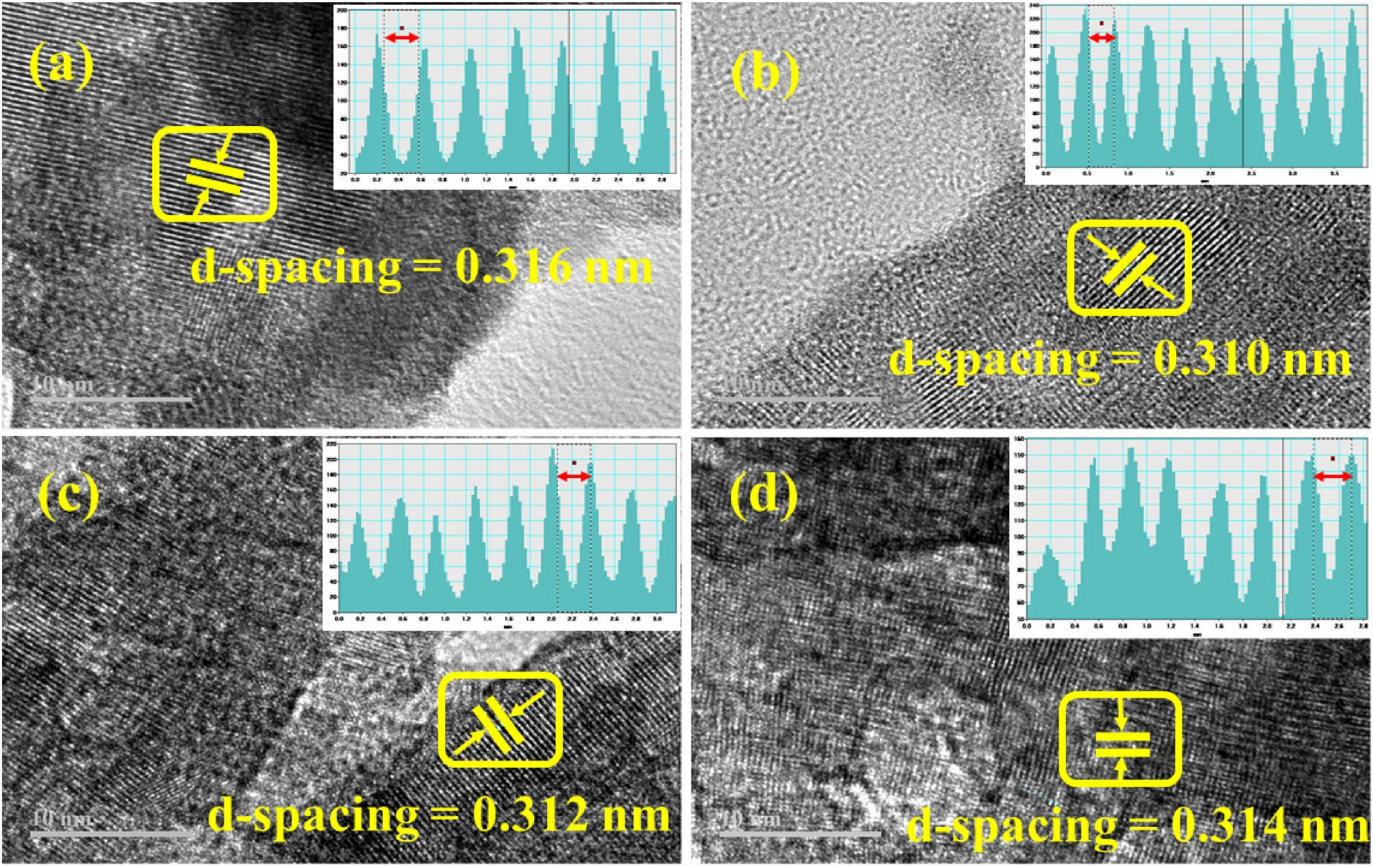
The interlayered d-spacing using HRTEM micrographs (**a**) Bi_2_WO_6_ (**b**) PVP-doped Bi_2_WO_6_ (**c**) 2% g-C_3_N_4_/PVP-doped Bi_2_WO_6_ (**d**) 4% g-C_3_N_4_/PVP-doped Bi_2_WO_6_ NRs.

The interlayer d-spacing of pristine and g-C_3_N_4_/PVP-doped Bi_2_WO_6_ NRs have been measured utilizing the HR-TEM micrograph with the assistance of Gatan digital software seen in Fig. 5a–d. The d-spacing for pristine and g-C_3_N_4_/PVP-doped Bi_2_WO_6_ NRs have been measured as 0.316, 0.310, 0.312, and 0.314 nm, respectively. The results obtained are consistent with the XRD crystallographic plane (131).

**Figure 6:**
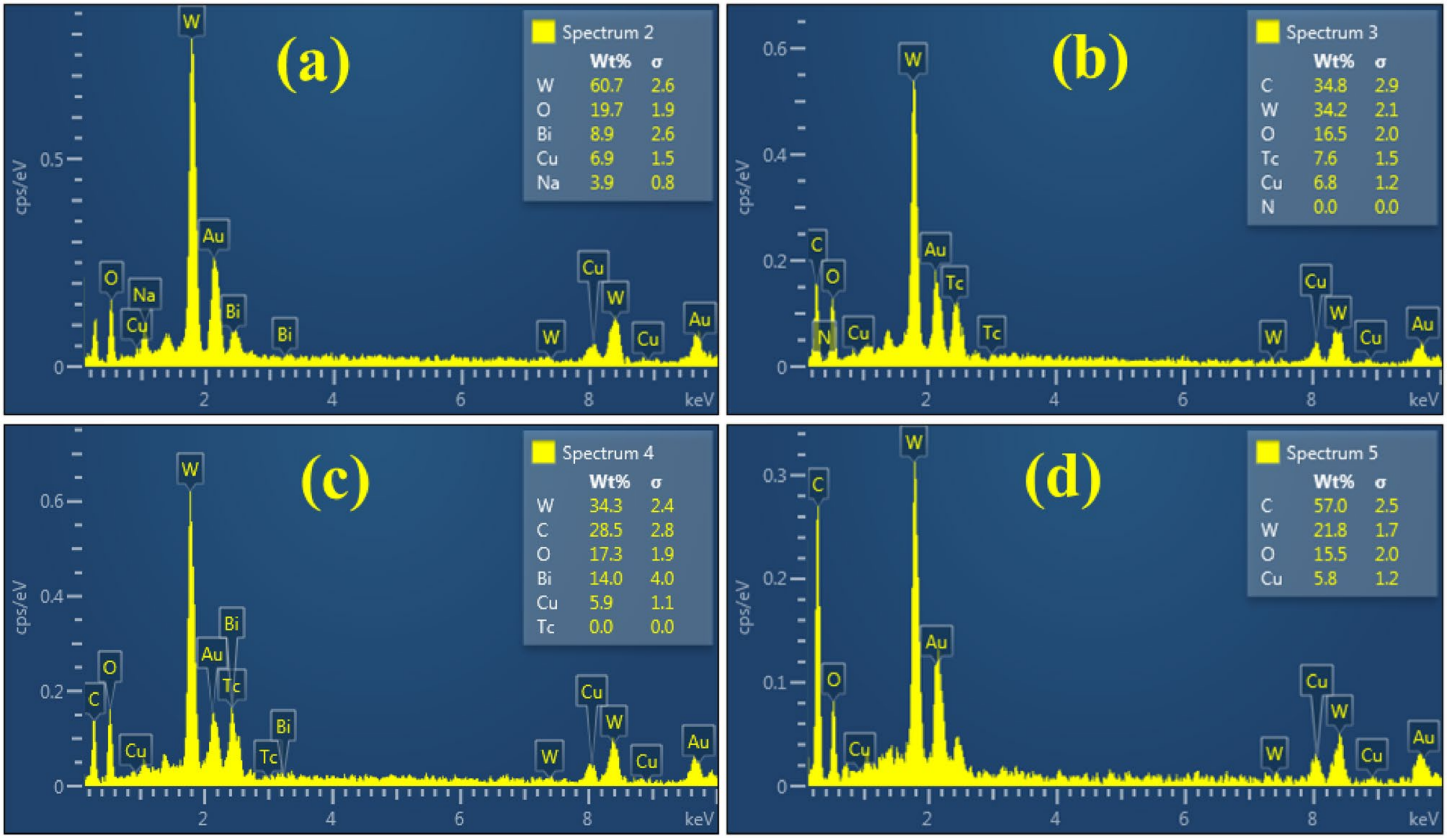
EDS analysis of (**a**) Bi_2_WO_6_ (**b**) PVP-doped Bi_2_WO_6_ (**c**) 2% g-C_3_N_4_/PVP-doped Bi_2_WO_6_ NRs (**d**) 4% g-C_3_N_4_/PVP-doped Bi_2_WO_6_ NRs.

EDS analysis has been utilized to validate the presence of bismuth, oxygen, and tungsten in the synthesized material. The elemental composition of pristine and g-C_3_N_4_/PVP-doped Bi_2_WO_6_ NRs has been investigated using EDS analysis displayed in Fig. 6a–d. The EDS analysis was used to investigate the elemental distribution to establish whether or not there is any additional interfacial contact. The appearance of significant peaks of the elements Bi, O, and W in the nanopowders provide conclusive evidence that Bi_2_WO_6_ NRs presented in the sample. The presence of carbon (C) peaks attributed to the PVP was successfully integrated. Some additional peaks appeared that are sodium (Na), copper (Cu), and gold (Au)^45^. The sample had a gold coating sprayed on it to reduce the charging impact of the sample so that the peaks of Au appeared in the spectrum. The peaks of Na have been found in the EDS analysis assigned to the NaOH used to maintain the pH of synthesized NRs. The appearance of the Cu peak can be attributed to the presence of Cu tape in the sample holder. Possible evidence of contamination includes the appearance of new peaks of technetium (Tc).

**Figure 7:**
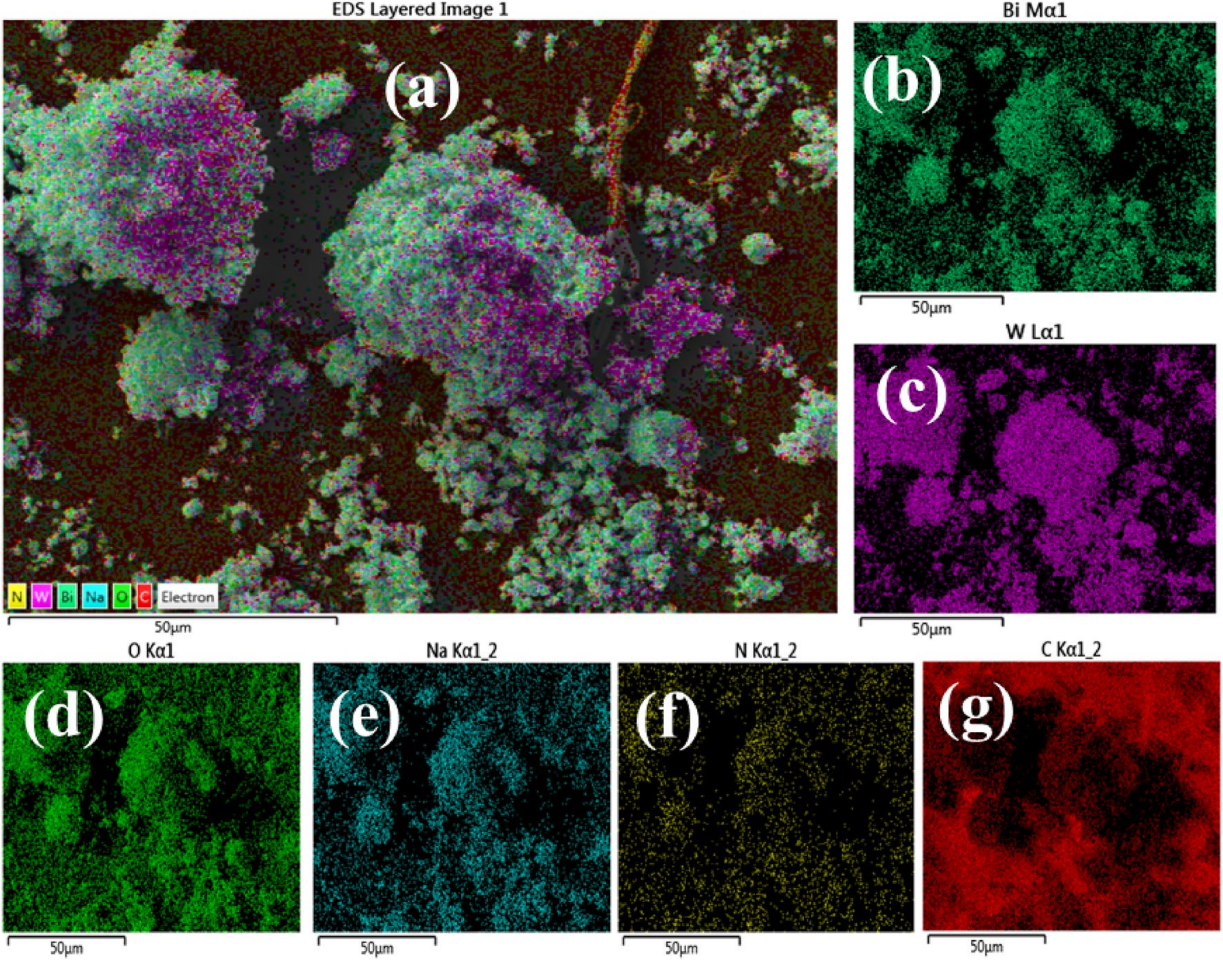
(**a**) Mapping analysis of synthesized NRs, (**b**) Bi, (**c**) W, (**d**) O, (**e**) Na, (**f**) N and (**g**) C.

Mapping analysis has been used to detect the presence of elements in the synthesized samples. The elemental mapping analysis of pristine Bi_2_WO_6_ and (2 and 4%) g-C_3_N_4_/PVP have been represented in Fig. 7a–g, which indicates the presence of W, O, and Bi in the pure sample after incorporation of g-C_3_N_4_/PVP indicate the appearance of C and N. The presence of sodium (Na) in the synthesized NRs, plays a role in both the regulation of pH and the formation of precipitates^45^.

**Figure 8:**
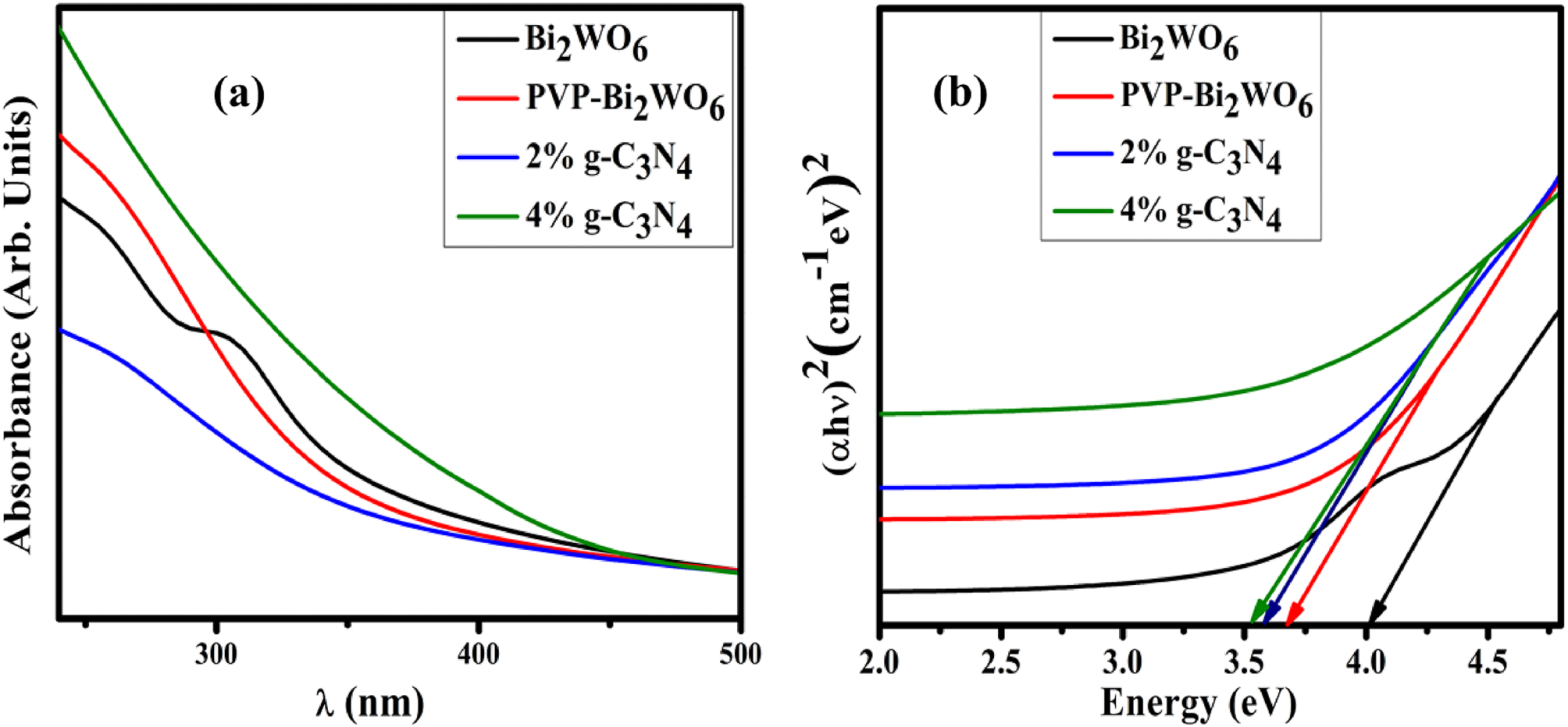
(**a**) UV–Vis absorption spectra (**b**) energy band gap calculation of pristine and g-C_3_N_4_/PVP-doped Bi_2_WO_6_ NRs.

Ultra-visible absorption spectroscopy was utilized to examine the optical absorption properties and the band gap in the 240–500 nm wavelength range. The optical properties of synthesized pristine and g-C_3_N_4_/PVP-doped Bi_2_WO_6_ NRs were indicated in Fig. 8a. The pristine sample exhibits an absorption peak at 305 nm^46^. Upon doping with PVP and g-C_3_N_4,_ absorption peaks shift gradually toward longer wavelengths indicating a redshift. Absorption spectra were employed for pure and doped samples to compute the band gap energy (Eg). The band gap was calculated via Tauc’s plot method using the extracted data from UV–Visible spectra can be represented in Fig. 8b. The energy band gap of pure Bi_2_WO_6_ is 4.0 eV. The band gap decreased from 4.0 to 3.5 eV on doping, as evident by the absorption spectra's redshift.

**Figure 9:**
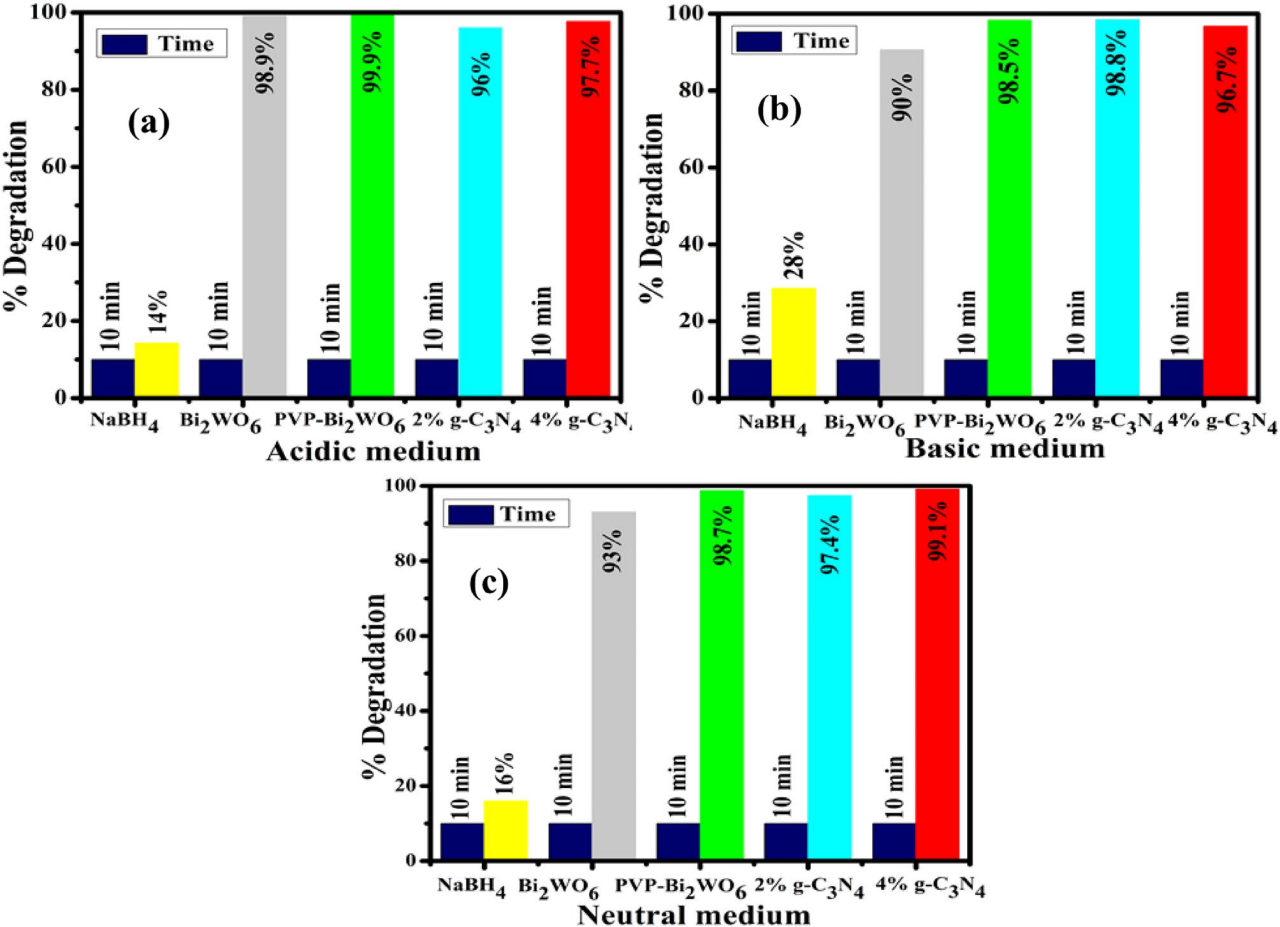
Catalytic activity of pristine and (2 and 4%) g-C_3_N_4_/PVP-doped Bi_2_WO_6_ NRs in (**a**) acidic medium (**b**) basic medium and (**c**) neutral medium.

Catalytic activity (CA) of pristine and (2 and 4%) g-C_3_N_4_/PVP-doped Bi_2_WO_6_ NRs for the degradation of RhB dye in the presence of NaBH_4_ at different pH values was measured using UV–vis spectrophotometer. The CA of synthesized catalysts depends on the pH, which is crucial in determining the extent of degraded dyes. Dye pollutants have been typically discharged at different pH levels. 0.5 M solution of H_2_SO_4_ was dissolved with RhB dye to maintain the pH of solution ∼2.5. The samples yield significant results as 98.9%, 99.9%, 96%, and 97.7% degradation were observed for 10 min. The catalytic degradation decreased with the incorporation of g-C_3_N_4_/PVP in the acidic dye solution because the redox reaction did not occur between the dye and the catalyst. In a basic medium, a 0.5 M solution of NaOH was utilized to achieve the desired pH∼12 in the dye solution. The findings demonstrate that the basic medium exhibited the highest rate of dye degradation, with recorded degradation percentages of 90%, 98.5%, 98.8%, and 96.7% for pristine and doped samples, respectively, after a 10-min duration. The catalytic degradation increased by incorporating a doped sample up to the optimum amount because of the redox reaction between the dye and the synthesized materials. In a neutral medium at pH ~ 7 results measured as 93%, 98.7%, 97.4%, and 99.1% for pristine and doped NRs respectively can be represented in Fig. 9a–c. The degradation or reduction of the potentially hazardous dye is triggered when a catalyst is added to the mixture of oxidizing and reducing agents. Surprisingly, an abundance of BH_4_^−^ donor species in the solution increases adsorption, whereas the transfer of electrons from the donor species to the accepter species through an oxidation and reduction reaction is required for the CA. The enhanced rate of reactions and attainment of the maximum degrading efficiency can be attributed to the particle's comparatively large surface area to its overall size^47^. The degradation of RhB was observed for all of the samples, demonstrating an increase in degradation efficiency for both pristine and doped samples due to the availability of active sites to adsorb. However, upon higher concentration doping, a decrease in the efficiency of RhB was observed due to an imbalance between the dye molecules and the nanocatalyst active site availability, which caused a weak adsorption capacity on the surface of the nanocatalyst. In general, the larger the surface area of the catalyst or the smaller its size, the more active sites it can provide to boost its CA. The enhanced efficiency of the catalyst can be attributed to the microporosity, which hinders the diffusion of the reactant into the active sites. Increasing the surface area of the catalyst leads to higher catalytic activity, potentially resulting from the production of a porous structure with a larger surface area^48^.

**Figure 10:**
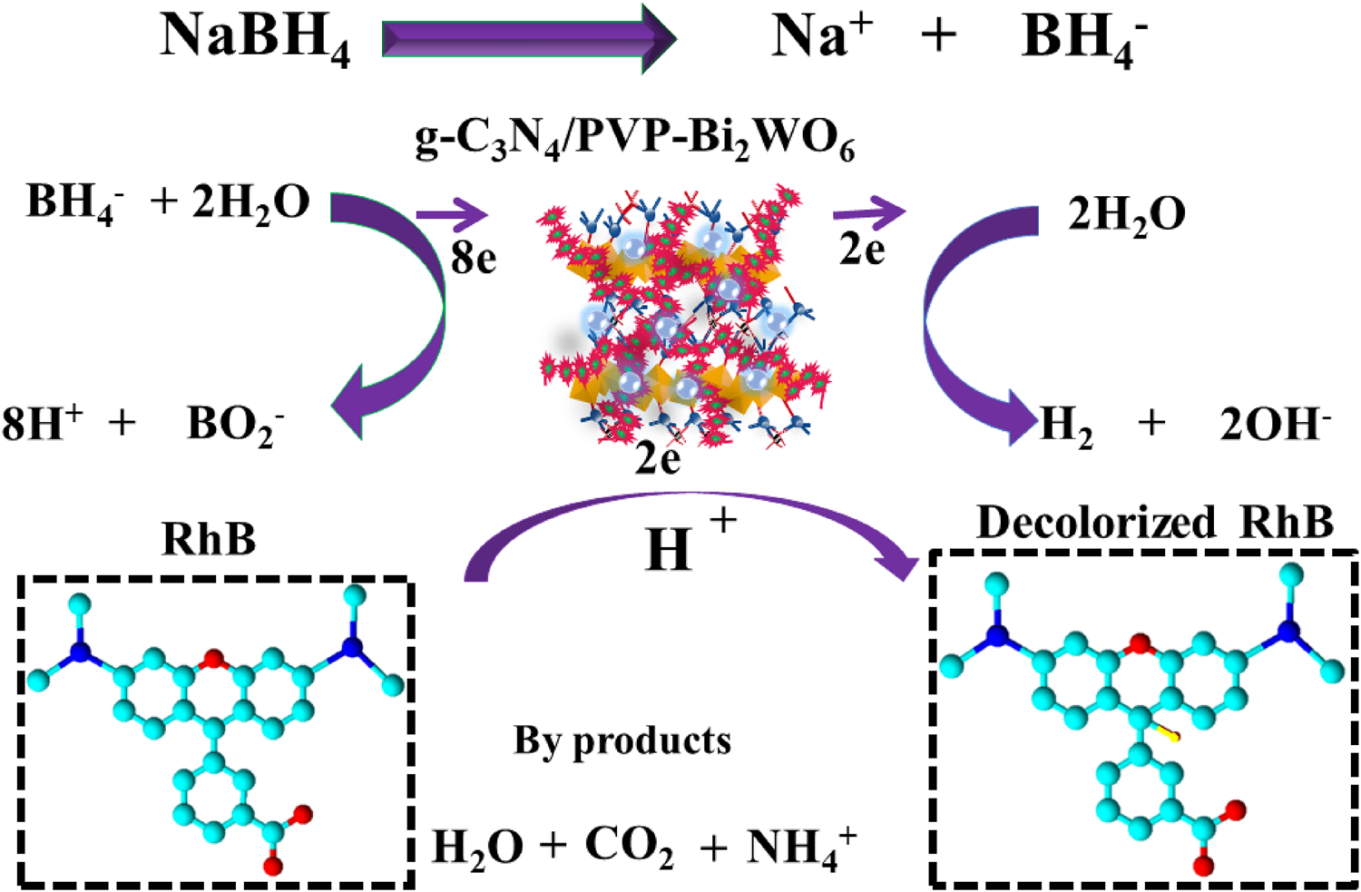
Catalytic mechanism for degradation of RhB dye.

The catalytic mechanism is depicted in Fig. 10, where the reducing agent and nanocatalyst are key factors in the degradation of RhB dye. The e-transfer from the reductant to the oxidant was involved in the redox reaction. As a result, the dye was broken down via electron absorption. The reaction was prolonged and less effective in the presence of NaBH_4_. To address these issues, nanocatalysts were incorporated into oxidation–reduction reactions. The catalyst acts as an electron relay by transferring electrons from NaBH_4_ to RhB, while NaBH_4_ acts as a reducing agent for the degradation of RhB. NRs increased the adsorption of BH_4_^−^ ions alongside dye molecules through many active sites, resulting in dye degradation that was faster and more efficient^49^.

The agar well diffusion method was used to investigate the in vitro antimicrobial activity of pristine and (2, 4%) g-C_3_N_4_/PVP-doped Bi_2_WO_6_ NRs by measuring inhibition zones. NRs possess antimicrobial activity that is effective against various strains of bacteria. Inhibition zones were statistically significant against *E. coli* found at low and high concentrations for pristine and doped Bi_2_WO_6_, respectively. The measurement of the inhibition zone for *E. coli* was compared with negative control, which consisted of DIW and had an inhibition zone evaluation of 0 mm, and positive control, which consisted of ciprofloxacin and had an inhibition zone measurement of 8.65 mm. Both the negative control and the positive control were used as comparisons. The pristine specimen produced low inhibition zones throughout the measurements when it was present in low quantities. However (2, 4%) g-C_3_N_4_/PVP-doped Bi_2_WO_6_ NRs were present in both high and low concentrations, it produced a large number of inhibition zones. In addition, the concentration of doped material is directly related to the zone of inhibition as the dopant concentration increased, which led to an expansion of the zone area. The inhibition zone measured between 1.35 and 2.45 mm in *E. coli* at low doses and between 3.25 and 4.55 mm in *E. coli* at high doses, as shown in Table 1.

**Table 1:** Antibacterial potential of Bi_2_WO_6_, and (2, 4%) g-C_3_N_4_/PVP-doped Bi_2_WO_6_.

Samples	0.5 mg/50 µL	1.0 mg/50 µL	Ciprofloxacin	DWI
*E. coli* inhibition zone (mm)				
Bi_2_WO_6_	1.35	3.25	8.65	0
PVP-doped Bi_2_WO_6_	1.75	3.65	8.65	0
2% g-C_3_N_4_/PVP-doped Bi_2_WO_6_	2.05	4.15	8.65	0
4% g-C_3_N_4_/PVP-doped Bi_2_WO_6_	2.45	4.55	8.65	0

**Figure 11:**
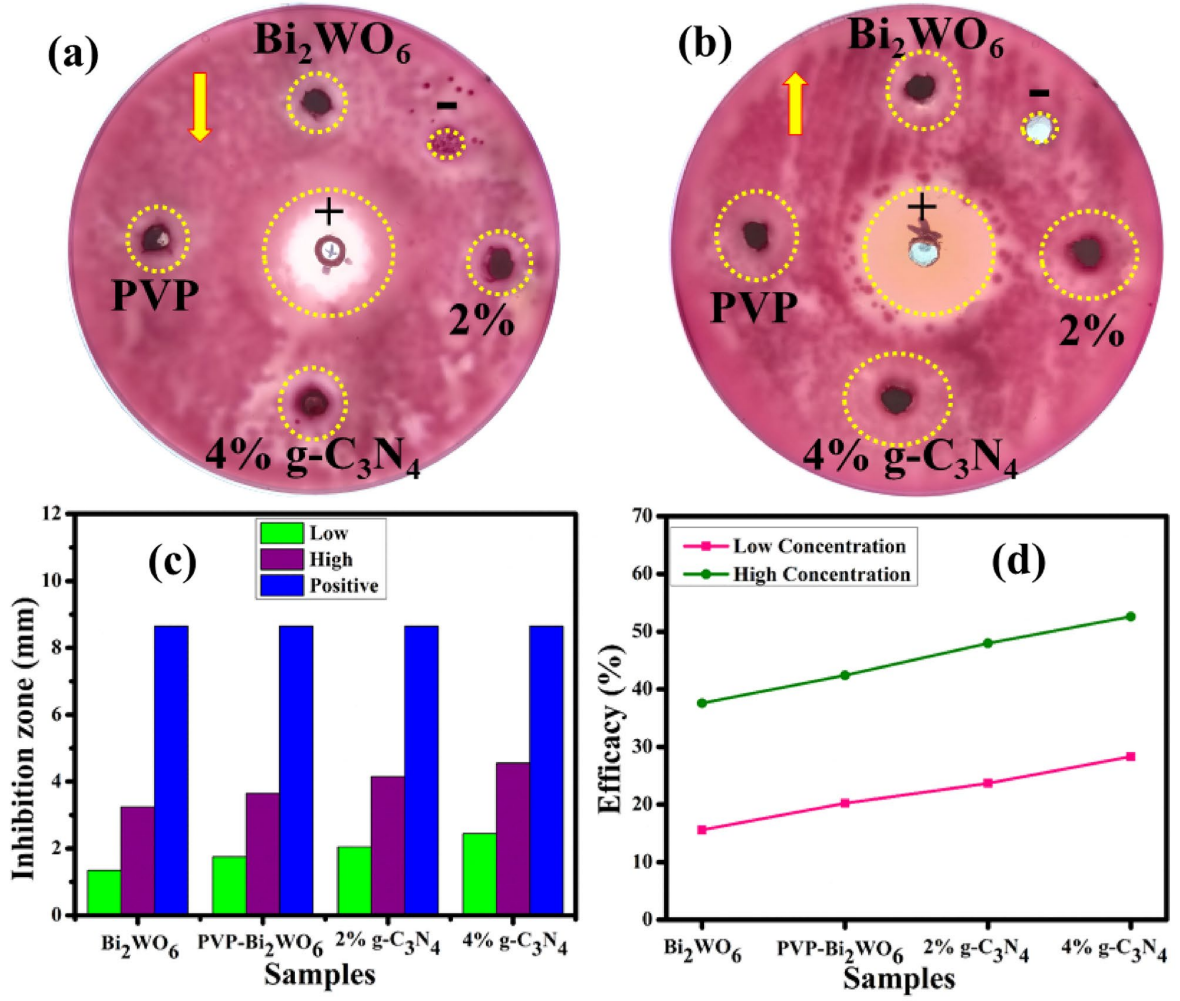
Photographic evidence and graphical representation of inhibition zones (**a**) low concentration, (**b**) high concentration, (**c**) comparative study of inhibition zones and (**d**) % age efficiency of pristine and g-C_3_N_4_/PVP-doped Bi_2_WO_6_ for *E. coli.*

The photographic evidence of antimicrobial activity for low and high concentration of synthesized materials can be represented in the Fig. 11a,b. The graphical representation of the inhibition zone measurement for antimicrobial analysis of pristine and g-C_3_N_4_/PVP-doped Bi_2_WO_6_ NRs for high and low inhibition zones compared to ciprofloxacin is shown in Fig. 11c. The % age efficacy for *E. coli* was calculated by dividing the sample's inhibition zone by the positive control inhibition area, which increased effectiveness at low and high concentrations by 15.60–28.32% and 37.57–52.60%, respectively, as represented in Fig. 11d.

The antimicrobial activity is caused by the interaction of OH groups in the PVP. Because of this increase in permeability and cytoplasmic leakage, the functional groups of components have the potential to form direct bonds with the phospholipids that make up the cell membrane^50^. NRs react with the cell wall and form a helical, disorganized spring on the cell wall and then enter the cell membrane, where they form interlinks with the structure of deoxyribonucleic acid (DNA) molecules^51^.

**Figure 12:**
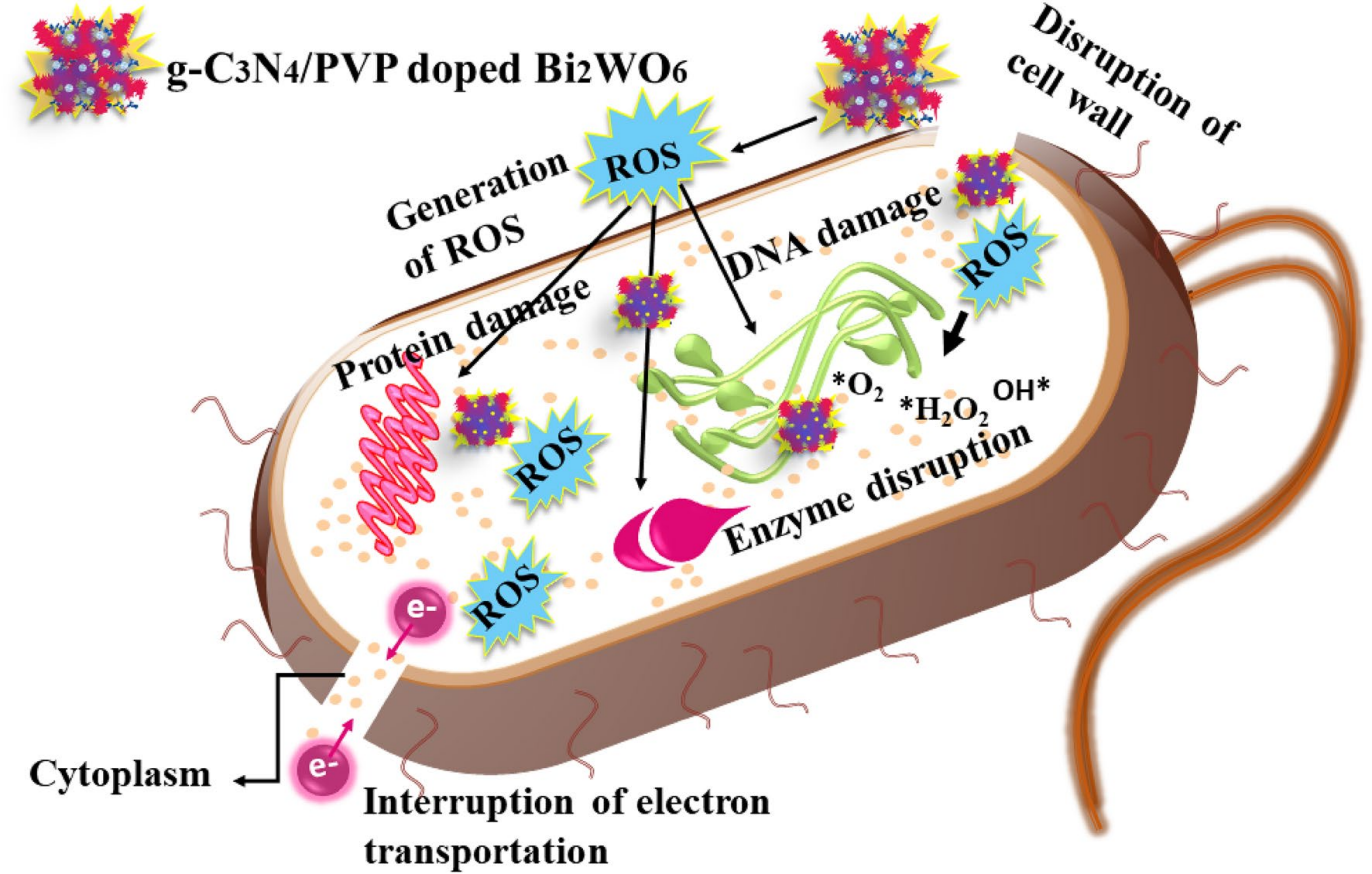
Schematic illustration of antibacterial mechanism.

The small particles actively produce reactive oxygen species (ROS), which cause the decomposition of the bacterial membrane and cytoplasmic contents, resulting in the bacteria's extinction^52^. The reaction that follows^53,54^ can be used to address ROS formation seen in Fig. 12.

$${\text{g - C}}_{{3}} {\text{N}}_{{4}} /{\text{PVP - doped Bi}}_{{2}} {\text{WO}}_{{6}} + {\text{ h}}\nu \left( {{\text{photon}}} \right) \, \to {\text{ h}}^{ + } \left( {{\text{hole}}} \right) \, + {\text{ e}}^{ - } \left( {\text{excited electron}} \right)$$

$${\text{e}}^{ - } + {\text{ O}}_{{2}} \to * {\text{O}}_{{2}}^{ - }$$

$${\text{h}}^{ + } + {\text{ H}}_{{2}} {\text{O }} \to {\text{ OH}}^{ - } + {\text{ H}}^{ + }$$

$$* {\text{O}}^{ - }_{{2}} + {\text{ H}}^{ + } \to * {\text{HO}}_{{2}}$$

$$* {\text{HO}}_{{2}} + {\text{ e}}^{ - } + {\text{ H}}^{ + } \to * {\text{H}}_{{2}} {\text{O}}_{{2}}$$

$$* {\text{H}}_{{2}} {\text{O}}_{{2}} + * {\text{O}}^{ - }_{{2}} \to * {\text{OH }} + {\text{ O}}_{{2}} + {\text{ OH}}^{ - }$$

The ability to eradicate bacteria is tied to the production of ROS, which would be influenced by a wide range of factors the crystallinity of the specimen, its surface area, and the quantity of oxygen-containing functional groups on its surface. These factors are responsible for the ability to kill bacteria^55^. The membrane of the bacteria is negatively charged, whereas the NRs have a positive charge. The interaction of a strong cationic (Bi^3+^) charge with a bacterial membrane, which results in increased bactericidal potential with increasing concentration of NRs, promotes lysis and bacterial cell collapse, ultimately resulting in bacterial cell death^56^.

**Figure 13:**
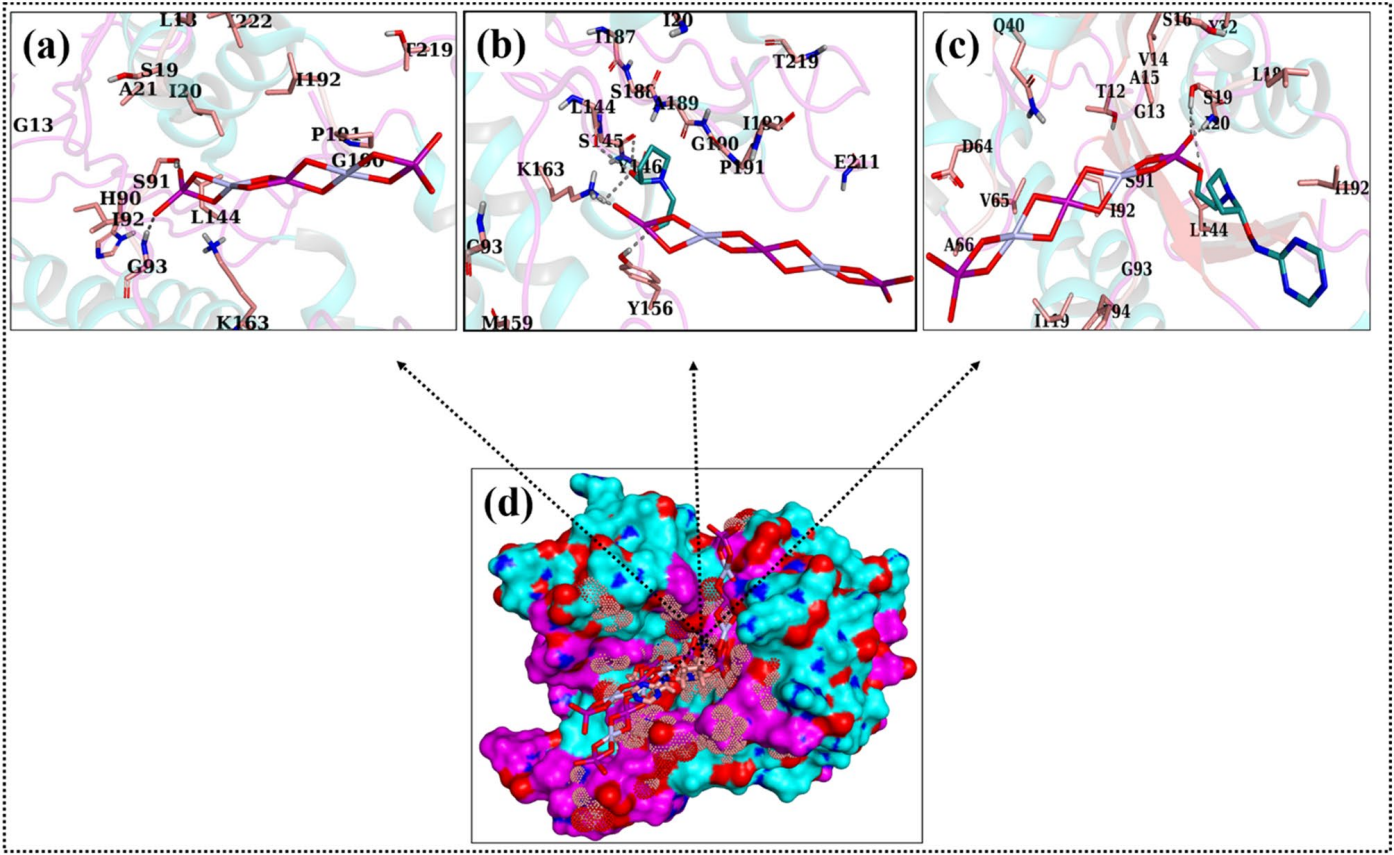
3D representation of the binding interaction pattern within the active site of FabI for Bi_2_WO_6_ (**a**), PVP-doped Bi_2_WO_6_ (**b**), g-C_3_N_4_/PVP-doped Bi_2_WO_6_ (**c**), and superimposed ligands (**d**).

The potential involvement of NMs as bactericidal agents has been widely documented; nevertheless, the reason for their potency remains a mystery that requires investigation. The disruption of many cellular processes by inhibiting enzyme targets has been regarded as a viable technique for discovering novel antibacterial drugs^57,58^. Computational methods, especially molecular docking, are useful for predicting the processes that could underlie certain biological functions. In the instance of FabI_*E.coli*_, these synthesized NRs showed strong binding interactions with critical amino acid residues of the active site where the best-docked complex was formed for Bi_2_WO_6_ NMs with a binding score of 4.24. As illustrated in Fig. 13a, two H-bond interactions, namely S91 and G93, were found. PVP-doped Bi_2_WO_6_ exhibited H-bond interactions with L144, S145, Y156, and K163, with a docking score of 5.26. Furthermore, the binding score found for g-C_3_N_4_/PVP-doped Bi_2_WO_6_ inside the active pocket of FabI_*E.coli*_ was rather high (7.0), indicating the participation of several H-bonds like S19 and I20, as shown in Fig. 13b–d.

**Figure 14:**
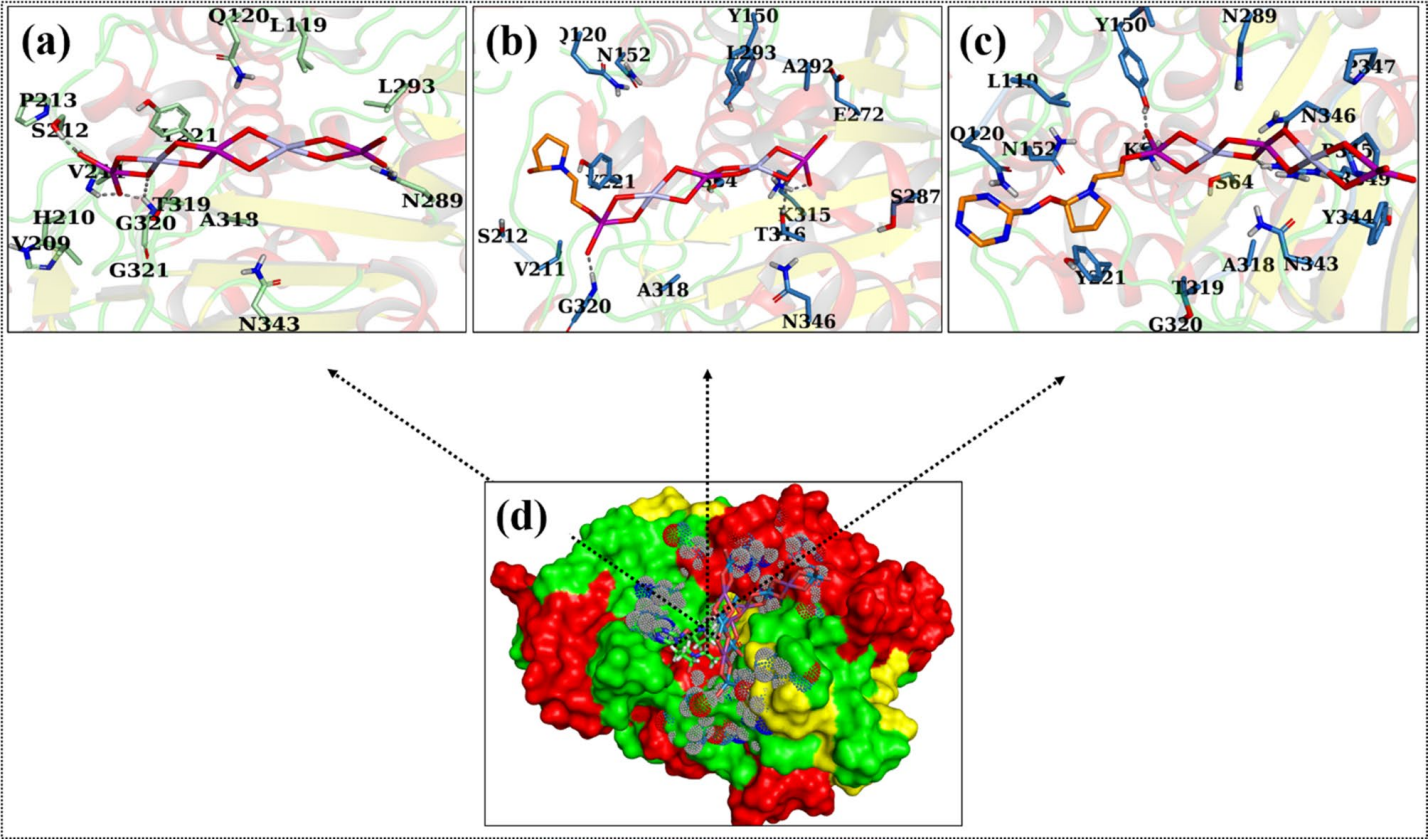
3D representation of the binding interaction pattern within the active site of β-lactamase for Bi_2_WO_6_ (**a**), PVP-doped Bi_2_WO_6_ (**b**), g-C_3_N_4_/PVP-doped Bi_2_WO_6_ (**c**), and superimposed ligands (**d**).

For β-lactamase_*E. Coli*_, binding affinity exhibited by g-C_3_N_4_/PVP-doped Bi_2_WO_6_ NRs was strongest with a binding score of 9.21. The H-bonding was observed with K67, Y150, N346, and R349. On the contrary, PVP-doped Bi_2_WO_6_ and Bi_2_WO_6_ NRs exhibited weaker binding affinities, as indicated by their binding scores of 8.67 and 4.13, respectively. Where three H-bonds (K315, T316, and G320) were observed for PVP-doped Bi_2_WO_6_ nanocomposites, while Bi_2_WO_6_ showed four H-bonds like V211, S212, N289, and G320, as shown in Fig. 14a–d.

## Conclusion

Novel Bi_2_WO_6_ and g-C_3_N_4_/PVP-doped Bi_2_WO_6_ nanorods (NRs) have been synthesized using the hydrothermal technique to investigate the catalytic and antibacterial activity as well as molecular docking analysis. Several structural and optical characterization techniques were employed to examine the characteristics of synthesized NRs. The XRD pattern endorsed the orthorhombic crystal structure of the pristine sample with different composition phases appearing as Bi_2_WO_6_ and Bi_2_W_2_O_9_ and improved crystallinity by incorporating dopants. The presence of functional groups in synthesized NRs and the stretching vibration of the Bi-O and W–O have been identified at 730 and 546 cm^–1^, respectively, using FTIR. SAED confirms the polycrystalline nature of synthesized NRs. TEM micrographs revealed the agglomeration of nanorods and particles overlapped by dopants. The interlayer d-spacing has been investigated using HR-TEM analysis and measured as 0.316, 0.310, 0.312, and 0.314 nm for Bi_2_WO_6_ and g-C_3_N_4_/PVP-doped Bi_2_WO_6_ NRs. The highest catalytic degradation of RhB can be measured as 99.9, 98.8, and 99.1% for acidic, basic, and neutral mediums. The inhibition zones for low and high NRs 2.45 and 4.55 mm can be used to investigate the antimicrobial activity against *E. coli* bacteria. The effectiveness of Bi_2_WO_6_, PVP-doped Bi_2_WO_6_, and g-C_3_N_4_/PVP-doped Bi_2_WO_6_ NRs as inhibitors of enoyl-[acylcarrierprotein] reductase (FabI) and the β-lactamase enzyme was evaluated, and their mechanisms of action were clarified by in silico research.

## Data Availability

The datasets used and analyzed during the current study available from the corresponding author on reasonable request.

## References

[1] Ismanto A, Hadibarata T, Widada S, Indrayanti E, Ismunarti DH, Safinatunnajah N, Kusumastuti W, Dwiningsih Y, Alkahtani J (2023). Groundwater contamination status in Malaysia: level of heavy metal, source, health impact, and remediation technologies. Bioprocess Biosyst. Eng..

[2] Mishra RK (2023). Fresh water availability and its global challenge. Br. J. Multidiscip. Adv. Stud..

[3] Sharma M, Behl K, Nigam S, Joshi M (2018). TiO2-GO nanocomposite for photocatalysis and environmental applications: A green synthesis approach. Vacuum..

[4] Zhai H, Huang L, Chen Z, Su Z, Yuan K, Liang G, Pan Y (2017). Chip-based molecularly imprinted monolithic capillary array columns coated GO/SiO2 for selective extraction and sensitive determination of rhodamine B in chili powder. Food Chem..

[5] Ostry V, Malir F, Toman J, Grosse Y (2017). Mycotoxins as human carcinogens—the IARC Monographs classification. Mycotoxin Res..

[6] Liang L, Cheng L, Zhang Y, Wang Q, Wu Q, Xue Y, Meng X (2020). Efficiency and mechanisms of rhodamine B degradation in Fenton-like systems based on zero-valent iron. RSC Adv..

[7] Santosham M, Chandran A, Fitzwater S, Fischer-Walker C, Baqui AH, Black R (2010). Progress and barriers for the control of diarrhoeal disease. Lancet..

[8] Munir N, Jahangeer M, Bouyahya A, El Omari N, Ghchime R, Balahbib A, Aboulaghras S, Mahmood Z, Akram M, Shah SMA, Mikolaychik IN, Derkho M, Rebezov M, Venkidasamy B, Thiruvengadam M, Shariati MA (2022). Heavy metal contamination of natural foods is a serious health issue: A review. Sustain..

[9] Fu W, Zhang W (2018). Microwave-enhanced membrane filtration for water treatment. J. Memb. Sci..

[10] Nasrollahzadeh M, Issaabadi Z, Sajadi SM (2018). Green synthesis of a Cu/MgO nanocomposite by: *Cassytha filiformis* L. extract and investigation of its catalytic activity in the reduction of methylene blue, congo red and nitro compounds in aqueous media. RSC Adv..

[11] Nasrollahzadeh M, Sajjadi M, Mohammad Sajadi S (2018). Biosynthesis of copper nanoparticles supported on manganese dioxide nanoparticles using *Centella asiatica* L. leaf extract for the efficient catalytic reduction of organic dyes and nitroarenes. Chin. J. Catal..

[12] Chao HJ, Xue D, Jiang W, Li D, Hu Z, Kang J, Liu D (2020). A low-voltage pulse electrolysis method for the degradation of anthraquinone and azo dyes in chloride medium by anodic oxidation on Ti/IrO2-RuO2-SnO2 electrodes. Water Environ. Res..

[13] El-Ashtoukhy ESZ, Fouad YO (2015). Liquid-liquid extraction of methylene blue dye from aqueous solutions using sodium dodecylbenzenesulfonate as an extractant. Alex. Eng. J..

[14] Salimi F, Emami SS, Karami C (2018). Removal of methylene blue from water solution by modified nano-boehmite with Bismuth. Inorg. Nano-Metal Chem..

[15] Bhattacharya, S., Saha, I., Mukhopadhyay, A., Chattopadhyay, D., & Chand, U. Role of nanotechnology in water treatment and purification: Potential applications and implications. *Int. J. Chem. Sci. Technol.* **3**, 59–64. https://www.academia.edu/download/51031736/Role_of_nanotechnology_in_water_treatmen20161223-22148-dx9shz.pdf (2013).

[16] Centi, G., Ciambelli, P., Perathoner, S., & Russo, P. Environmental catalysis: Trends and outlook. In Catal. Today, Elsevier, pp. 3–15. 10.1016/S0920-5861(02)00037-8 (2002).

[17] Das S, Chakraborty J, Chatterjee S, Kumar H (2018). Prospects of biosynthesized nanomaterials for the remediation of organic and inorganic environmental contaminants. Environ. Sci Nano..

[18] Mallikarjunaswamy C, Pramila S, Nagaraju G, Ramu R, Ranganatha VL (2021). Green synthesis and evaluation of antiangiogenic, photocatalytic, and electrochemical activities of BiVO4 nanoparticles. J. Mater. Sci. Mater. Electron..

[19] Deepakumari HN, Lakshmi Ranganatha V, Nagaraju G, Prakruthi R, Mallikarjunaswamy C (2022). Facile green synthesis of zirconium phosphate nanoparticles using Aegle marmelos: Antimicrobial and photodegradation studies. Mater. Today Proc..

[20] Pramila S, Ranganatha VL, Soundarya TL, Ramu R, Nagaraju G, Mallikarjunaswamy C (2022). Eco-mediated synthesis of visible active Bi2WO6 nanoparticles and its performance towards photocatalyst, supercapacitor, biosensor, and antioxidant activity. J. Clust. Sci..

[21] Lou Z, Lu C, Li X, Wu Q, Li J, Wen L, Dai Y, Huang B, Li B (2021). Constructing surface plasmon resonance on Bi2WO6 to boost high-selective CO2 reduction for methane. ACS Nano..

[22] Saison T, Chemin N, Chaneéac C, Durupthy O, Ruaux V, Mariey L, Maugeé F, Beaunier P, Jolivet JP (2011). Bi2O3, BiVO4, and Bi2WO 6: Impact of surface properties on photocatalytic activity under visible light. J. Phys. Chem. C..

[23] Koczkur KM, Mourdikoudis S, Polavarapu L, Skrabalak SE (2015). Polyvinylpyrrolidone (PVP) in nanoparticle synthesis. Dalt. Trans..

[24] Nathanael AJ, Seo YH, Oh TH (2015). PVP assisted synthesis of hydroxyapatite nanorods with tunable aspect ratio and bioactivity. J. Nanomater..

[25] Al-Saidi WA, Feng H, Fichthorn KA (2012). Adsorption of polyvinylpyrrolidone on Ag surfaces: Insight into a structure-directing agent. Nano Lett..

[26] Khan WH, Rathod VK (2014). Process intensification approach for preparation of curcumin nanoparticles via solvent-nonsolvent nanoprecipitation using spinning disc reactor. Chem. Eng. Process. Process Intensif..

[27] Ivanov AS, Miller E, Boldyrev AI, Kameoka Y, Sato T, Tanaka K (2015). Pseudo jahn-teller origin of buckling distortions in two-dimensional triazine-based graphitic carbon nitride (g-C3N4) sheets. J. Phys. Chem. C..

[28] Liu W, Wang M, Xu C, Chen S (2012). Facile synthesis of g-C3N4/ZnO composite with enhanced visible light photooxidation and photoreduction properties. Chem. Eng. J..

[29] Sinclair CG (1939). Bergey’s manual of determinative bacteriology. Am. J. Trop. Med. Hyg..

[30] Bauer AW, Kirby WM, Sherris JC, Turck M (1966). Antibiotic susceptibility testing by a standardized single disk method. Am. J. Clin. Pathol..

[31] Feuerstein A, Scuda N, Klose C, Hoffmann A, Melchner A, Boll K, Rettinger A, Fell S, Straubinger RK, Riehm JM (2022). Antimicrobial resistance, serologic and molecular characterization of E. coli isolated from calves with severe or fatal enteritis in Bavaria, Germany. Antibiotics..

[32] Weinstein MP, Lewis JS (2020). The clinical and laboratory standards institute subcommittee on Antimicrobial susceptibility testing: Background, organization, functions, and processes. J. Clin. Microbiol..

[33] Iwalokun BA, Ogunledun A, Ogbolu DO, Bamiro SB, Jimi-Omojola J (2004). In vitro antimicrobial properties of aqueous garlic extract against multidrug-resistant bacteria and Candida species from Nigeria. J. Med. Food..

[34] Naika HR, Lingaraju K, Manjunath K, Kumar D, Nagaraju G, Suresh D, Nagabhushana H (2015). Green synthesis of CuO nanoparticles using *Gloriosa superba* L. extract and their antibacterial activity. J. Taibah Univ. Sci..

[35] Haider A, Ijaz M, Imran M, Naz M, Majeed H, Khan JA, Ali MM, Ikram M (2020). Enhanced bactericidal action and dye degradation of spicy roots’ extract-incorporated fine-tuned metal oxide nanoparticles. Appl. Nanosci..

[36] Haider A, Ijaz M, Ali S, Haider J, Imran M, Majeed H, Shahzadi I, Ali MM, Khan JA, Ikram M (2020). Green synthesized phytochemically (zingiber officinale and allium sativum) reduced nickel oxide nanoparticles confirmed bactericidal and catalytic potential. Nanoscale Res. Lett..

[37] Drawz SM, Bonomo RA (2010). Three decades of β-lactamase inhibitors. Clin. Microbiol. Rev..

[38] Schiebel J, Chang A, Merget B, Bommineni GR, Yu W, Spagnuolo LA, Baxter MV, Tareilus M, Tonge PJ, Kisker C, Sotriffer CA (2015). An Ordered Water Channel in Staphylococcus aureus FabI: Unraveling the Mechanism of Substrate Recognition and Reduction. Biochemistry..

[39] Barelier S, Eidam O, Fish I, Hollander J, Figaroa F, Nachane R, Irwin JJ, Shoichet BK, Siegal G (2014). Increasing chemical space coverage by combining empirical and computational fragment screens. ACS Chem. Biol..

[40] Mehmood Z, Ikram M, Imran M, Shahzadi A, Haider A, Ul-Hamid A, Nabgan W, Haider J, Hayat S, Z, (2022). officinale-doped silver/calcium oxide nanocomposites: Catalytic activity and antimicrobial potential with molecular docking analysis. Process Biochem..

[41] Shahzadi I, Islam M, Saeed H, Haider A, Shahzadi A, Haider J, Ahmed N, Ul-Hamid A, Nabgan W, Ikram M, Rathore HA (2022). Formation of biocompatible MgO/cellulose grafted hydrogel for efficient bactericidal and controlled release of doxorubicin. Int. J. Biol. Macromol..

[42] Dutta V, Sonu S, Raizada P, Thakur VK, Ahamad T, Thakur S, Kumar Verma P, Quang HHP, Nguyen VH, Singh P (2022). Prism-like integrated Bi2WO6 with Ag-CuBi2O4 on carbon nanotubes (CNTs) as an efficient and robust S-scheme interfacial charge transfer photocatalyst for the removal of organic pollutants from wastewater. Environ. Sci. Pollut. Res..

[43] Mengting Z, Kurniawan TA, Yanping Y, Avtar R, Othman MHD (2020). 2D Graphene oxide (GO) doped p-n type BiOI/Bi2WO6 as a novel composite for photodegradation of bisphenol A (BPA) in aqueous solutions under UV-vis irradiation. Mater. Sci. Eng. C..

[44] Hao Q, Niu X, Nie C, Hao S, Zou W, Ge J, Chen D, Yao W (2016). A highly efficient g-C 3 N 4 /SiO 2 heterojunction: The role of SiO 2 in the enhancement of visible light photocatalytic activity. Phys. Chem. Chem. Phys..

[45] Mishra R, Jamshaid H, Militky J (2017). Basalt nanoparticle reinforced hybrid woven composites: Mechanical and thermo-mechanical performance. Fibers Polym..

[46] Li D, Yan P, Zhao Q, Bai X, Ma X, Xue J, Zhang Y, Liu M (2020). Synthesis of Bi2WO6/Bi2MoO6 heterostructured nanosheet and activating peroxymonosulfate to enhance photocatalytic activity. J. Inorg. Organomet. Polym. Mater..

[47] Roy H, Rahman TU, Khan MAJR, Al-Mamun MR, Islam SZ, Khaleque MA, Hossain MI, Khan MZH, Islam MS, Marwani HM, Islam A, Hasan MM, Awual MR (2023). Toxic dye removal, remediation, and mechanism with doped SnO2-based nanocomposite photocatalysts: A critical review. J. Water Process Eng..

[48] Zhang X, Hou F, Li H, Yang Y, Wang Y, Liu N, Yang Y (2018). A strawsheave-like metal organic framework Ce-BTC derivative containing high specific surface area for improving the catalytic activity of CO oxidation reaction. Microporous Mesoporous Mater..

[49] Tejwan N, Sharma A, Thakur S, Das J (2021). Green synthesis of a novel carbon dots from red Korean ginseng and its application for Fe2+ sensing and preparation of nanocatalyst. Inorg. Chem. Commun..

[50] Kasai D, Chougale R, Masti S, Chalannavar R, Malabadi RB, Gani R, Gouripur G (2019). An investigation into the influence of filler piper nigrum leaves extract on physicochemical and antimicrobial properties of chitosan/poly (Vinyl Alcohol) blend films. J. Polym. Environ..

[51] Czarnecki MA, Wojtków D (2008). Effect of varying water content on the structure of butyl alcohol/water mixtures: FT-NIR two-dimensional correlation and chemometric studies. J. Mol. Struct..

[52] Kannan K, Radhika D, Reddy KR, Raghu AV, Sadasivuni KK, Palani G, Gurushankar K (2021). Gd3+and Y3+co-doped mixed metal oxide nanohybrids for photocatalytic and antibacterial applications. Nano Express..

[53] Madona J, Sridevi C (2022). Surfactant assisted hydrothermal synthesis of MgO/g-C3N4 heterojunction nanocomposite for enhanced solar photocatalysis and antimicrobial activities. Inorg. Chem. Commun..

[54] Kessler A, Hedberg J, Blomberg E, Odnevall I (2022). Reactive Oxygen species formed by metal and metal oxide nanoparticles in physiological media—a review of reactions of importance to nanotoxicity and proposal for categorization. Nanomaterials..

[55] Karthik K, Dhanuskodi S, Gobinath C, Prabukumar S, Sivaramakrishnan S (2019). Fabrication of MgO nanostructures and its efficient photocatalytic, antibacterial and anticancer performance. J. Photochem. Photobiol. B Biol..

[56] Kannan K, Radhika D, Nesaraj AS, Kumar Sadasivuni K, Reddy KR, Kasai D, Raghu AV (2020). Photocatalytic, antibacterial and electrochemical properties of novel rare earth metal oxides-based nanohybrids. Mater. Sci. Energy Technol..

[57] Ikram M, Hafeez I, Naz M, Haider A, Naz S, Ul-Hamid A, Haider J, Shahzadi A, Imran M, Nabgan W, Ali S (2022). Highly efficient industrial dye degradation, bactericidal properties, and in silico molecular docking analysis of Ag/cellulose-doped CuO nanostructures. ACS Omega..

[58] Shujah T, Shahzadi A, Haider A, Mustajab M, Haider AM, Ul-Hamid A, Haider J, Nabgan W, Ikram M (2022). Molybdenum-doped iron oxide nanostructures synthesized via a chemical co-precipitation route for efficient dye degradation and antimicrobial performance: in silico molecular docking studies. RSC Adv..

